# Visual Assessment and Information Effects on Consumer Acceptance of Insect-Based Foods: The Role of Attitudes, Knowledge, and Sociodemographics

**DOI:** 10.3390/foods15101703

**Published:** 2026-05-12

**Authors:** Alessandra Verardi, Paola Sangiorgio, Olga Mileti, Mariateresa Chiodo, Noemi Baldino, Simona Errico

**Affiliations:** 1 Laboratory Regenerative Circular Bioeconomy, Italian National Agency for New Technologies, Energy and Sustainable Economic Development, Trisaia Research Centre, 75026 Rotondella, Italy; paola.sangiorgio@enea.it (P.S.); simona.errico@enea.it (S.E.); 2 Department of Computer Engineering, Modelling, Electronics and Systems (DIMES), University of Calabria (UNICAL), Via Pietro Bucci, 87036 Arcavacata di Rende, Italy; o.mileti@dimes.unical.it (O.M.); mariateresa.chiodo@unical.it (M.C.); noemi.baldino@unical.it (N.B.)

**Keywords:** insect-based foods, consumer acceptance, consumer attitudes, consumer knowledge, food neophobia, visual evaluation, sociodemographic factors

## Abstract

This study investigates consumer acceptance of insect-based foods, focusing on changes in visual evaluation after information disclosure and the influence of sociodemographic, attitudinal, and knowledge-related factors. An online survey among Italian consumers (n = 350) assessed the visual attractiveness of a cupcake containing 10% *w*/*w Tenebrio molitor* flour before (PRE) and after (POST) disclosure of the insect ingredient. Attractiveness decreased from 2.6 to 2.0, with 79% of POST evaluations in the lowest appeal categories. Women expressed more negative POST ratings and experienced a larger decline in attractiveness (Δ = POST − PRE), indicating greater sensitivity to information disclosure than men. The change in attractiveness (Δ) was linked to psychological variables: negative attitudes showed moderate negative correlations with Δ (r ≈ −0.3 to −0.6), whereas higher knowledge of regulatory, nutritional, and environmental aspects showed positive correlations (r ≈ +0.3 to +0.7), mitigating the decrease. Principal Component Analysis revealed two latent dimensions: PC1 (61.6%), representing an attitudinal continuum from aversion to acceptance, and PC2 (33.3%), reflecting differences in awareness. Respondents with higher PC1 and PC2 scores showed attenuated Δ values, indicating greater resilience to the disclosure effect. Overall, findings highlight a gap between visual familiarity and acceptance, shaped by emotions, knowledge, and gender-specific sensitivities.

## 1. Introduction

In March 2026, the global population reached approximately 8.28 billion people (Worldometers, 2026), marking a substantial increase from 5 billion in 1986 and 7.7 billion in 2019 [1,2]. This growth is expected to continue over the coming decades, with projections estimating 10.1–10.6 billion people by 2050 and up to 12.7–15.6 billion by 2100 [3]. Such demographic expansion will place increasing pressure on global food systems. According to the Food and Agriculture Organization, achieving the Sustainable Development Goal of zero hunger by 2030 will require a substantial increase in food production compared with current levels [4].

Ensuring adequate food supply while maintaining environmental sustainability represents a major challenge for modern food systems. Conventional livestock production, which provides a large share of global protein intake, is associated with significant environmental impacts, including greenhouse gas emissions, extensive land and water requirements, and biodiversity loss [5,6].

Among the proposed alternatives, edible insects have attracted increasing scientific and industrial attention. Insects have long been recognized as a potential solution to future food shortages and are characterized by highly efficient feed conversion rates, reduced resource requirements, lower environmental impact, and the possibility of being reared on organic side streams, thereby contributing to waste reduction and circular economy approaches [5,7,8,9]. From a nutritional perspective, edible insects provide high-quality proteins, essential amino acids, fatty acids, vitamins, and micronutrients such as iron and zinc, making them a valuable component of diversified and sustainable diets [10,11,12].

Insect consumption is a long-established dietary practice in numerous cultures worldwide, where insects are incorporated into traditional dishes [7,13,14]. In particular, entomophagy is widely documented in regions such as Southeast Asia, sub-Saharan Africa, and Latin America, where insects are consumed as part of everyday diets. In these contexts, insects are incorporated into a variety of traditional dishes, including fried crickets and grasshoppers in Southeast Asia, mopane worms in Southern Africa, and chapulines in several regions of Mexico [7,8,14,15].

The commercialization of insect-based foods (IBFs) within the European Union is relatively recent and currently restricted to a small number of species authorized under the Novel Food Regulation, including *Tenebrio molitor* (TM, yellow mealworm) [16], *Locusta migratoria* [17], *Acheta domesticus* [18], and *Alphitobius diaperinus* [18]. In response to regulatory approval and growing interest in sustainable protein alternatives, recent years have seen the development of a range of novel insect-based products designed for Western markets, such as protein bars, snacks, pasta, baked goods, and hybrid formulations in which insect flour is incorporated into familiar food matrices [19,20]. Despite their long-standing use in many regions of the world, IBFs still face limited acceptance in Western societies [21,22]. Cultural barriers, food neophobia, and feelings of disgust represent major obstacles [15,23,24]. Consequently, understanding the determinants of consumer attitudes and identifying strategies capable of positively influencing public perception are essential for promoting the wider adoption of edible insects in future food systems. Beyond immediate affective reactions, food neophobia and neophilia have also been linked to memory- and experience-based processes, whereby prior exposure, cultural learning, and emotionally salient experiences shape expectations toward novel foods and influence acceptance or rejection [25,26,27,28,29].

Previous studies have shown that incorporating insect ingredients into processed foods, where insects are not visually recognizable, may reduce negative reactions and increase consumers’ willingness to try such products. At the same time, information and communication strategies have been identified as key factors influencing consumer perception.

Although taste is a primary determinant of food choice [30], the first sensory interaction with food frequently occurs through visual perception. Color, presentation, and structure influence expectations of taste, quality, and safety, and can significantly affect consumer acceptance [31,32,33,34,35,36,37,38]. These effects are particularly relevant when individuals are exposed to novel foods, as visual familiarity can reduce neophobia and increase willingness to try unfamiliar products [25,26]. Consistent with these findings, a recent report by the International Platform of Insects for Food and Feed (IPIFF) shows that European consumers display higher acceptance when insects are incorporated as ingredients (e.g., flour) in familiar food products such as bars, pasta, and snacks, compared to whole insects [20].

However, much of the existing research on consumer acceptance of IBFs has focused on tasting experiments or hypothetical willingness-to-try scenarios. Although these approaches provide valuable insights, they often combine sensory evaluation with cognitive judgments, making it difficult to isolate the specific influence of visual perception and information disclosure. In particular, limited studies have examined how consumers evaluate IBFs based solely on visual cues and how their perceptions change after being informed of the presence of insect ingredients [39].

Based on the literature reviewed above, the present study adopts an exploratory pre–post approach in which information disclosure is conceptualized as a cognitive trigger acting on an already formed visual evaluation, rather than within a formal expectation-confirmation or framing framework. This approach allows the examination of how ingredient information reshapes pre-existing visual perceptions under controlled conditions.

Accordingly, the present study was guided by the following hypotheses: the initial visual evaluation of a familiar baked product would result in neutral to moderately positive ratings; disclosure of the presence of insect flour would significantly reduce perceived attractiveness; and this change in perception would be influenced by individual factors, including sociodemographic characteristics as well as attitudinal and cognitive variables.

Therefore, the objective of this study was to investigate how visual evaluation of a familiar insect-based food product changes following ingredient disclosure, and how this change is modulated by sociodemographic characteristics, consumer attitudes, and prior knowledge.

## 2. Materials and Methods

### 2.1. Survey Design

The study was designed as a cross-sectional survey including a pre–post evaluation, in which participants assessed the same product before and after receiving information about its composition.

An online survey was conducted in Italy between October and November 2025 to investigate consumer acceptance of IBFs, with a specific focus on visual perception and the effect of information on product evaluation. The survey was administered via Google Forms and distributed via a non-probability snowball sampling approach. The non-probability snowball sampling approach was adopted in accordance with the exploratory nature of the study. The primary aim was not to obtain a statistically representative sample of the Italian population, but to investigate underlying perceptual, attitudinal, and cognitive mechanisms associated with the visual evaluation of insect-based foods and the effect of information disclosure. Similar non-probability sampling strategies are commonly employed in online consumer research on novel or emerging foods, including insect-based products, where the focus lies on identifying psychological drivers of acceptance rather than estimating population-level prevalence [40,41,42]. Moreover, snowball-based recruitment is widely recognized as an effective approach for engaging respondents willing to participate in surveys addressing unfamiliar or potentially controversial food topics in an online context [43]. Initial participants were recruited via personal social networks, institutional mailing lists, and online communication channels, and were subsequently invited to share the survey with their contacts, allowing for rapid data collection across different regions of Italy. A total of 350 Italian participants completed the questionnaire, which was distributed through multiple channels, including social media platforms (e.g., Facebook, WhatsApp), institutional mailing lists, and dedicated online pages. The resulting sample size is comparable to, and in several cases larger than, that reported in other online survey-based studies investigating consumer acceptance of insect-based and novel foods [41,44,45].

Participation was voluntary and anonymous. It was possible to withdraw at any time by closing the browser and not submitting the form. Before accessing the questionnaire, respondents were provided with an electronic informed consent form describing the purpose of the study, data confidentiality, and the voluntary nature of participation. Only participants who provided consent were allowed to proceed.

### 2.2. Questionnaire Design

Before accessing the questionnaire, participants were provided with information about the study objectives and were required to provide informed consent.

The questionnaire consisted of 10 questions and required approximately 5 min to complete (Supplementary Materials, Section S1). It was designed in 5 sections (Table 1) to ensure clarity, simplicity, and a logical flow. All questionnaire sections were presented in a fixed and identical order for all participants, without randomization.

All responses were anonymous and treated as confidential. The results were analyzed in aggregated form to prevent the identification of individual participants. Raw data access was securely stored, and access was restricted to authorized and identified personnel of the Italian National Agency for New Technologies, Energy and Sustainable Economic Development (ENEA).

The attitudinal (ATT) items were selected “*a priori*” based on established literature on consumer acceptance of insect-based foods and novel foods. Previous research consistently conceptualizes attitudes toward entomophagy as a multidimensional construct encompassing affective responses (e.g., discomfort and disgust), cognitive evaluations (e.g., perceived safety, health-related concerns, and expectations of future diffusion), and behavioral intentions (e.g., curiosity and willingness to try). Accordingly, the ATT item set was intentionally designed to capture different psychological facets of consumer attitudes toward insect-based foods rather than to represent a single latent trait. Similar item formulations have been widely employed in prior studies addressing psychological barriers and drivers of acceptance of insect-based foods and other novel food products.

The knowledge-related (KNO) items were developed to assess respondents’ prior awareness of regulatory, nutritional, and environmental aspects of insect-based foods, in line with literature highlighting the role of objective knowledge in shaping consumer responses to novel and sustainable food products.

Before data collection, the questionnaire underwent an internal expert review by researchers with experience in food science and consumer research to ensure content relevance, clarity, and coherence with the study objectives.

### 2.3. Cupcake Preparation

The bakery product used as a visual stimulus in the survey (cupcake) was developed at the University of Calabria (UNICAL) using TM flour provided by the Italian National Agency for New Technologies, Energy and Sustainable Economic Development (ENEA). All other ingredients were purchased from a local supermarket and are butter (Despar, Casalecchio di Reno, Italy), sunflower oil (Olearia De Santis S.p.A., Bitonto, Italy), eggs (Delizie del sole, San Martino Buon Albergo, Italy), sugar (Dolciando, San Martino Buon Albergo, Italy), 00 wheat flour (Tre mulini, Bologna, Italy), UTH whole milk (Land, Castenedolo, Italy), and baking powder (Dolciando).

Different formulations were prepared, including a standard recipe (C) prepared with butter, a recipe prepared with sunflower oil (CO) and CO’s variants enriched with TM flour at two levels, 5% *w*/*w* and 10% *w*/*w*, CO5%TM and CO10%TM, respectively, in line with current regulatory limits [46]. Table 2 reports on the composition of all recipes.

The ingredients were mixed using a planetary mixer (KitchenAid, Benton Harbor, MI, USA) according to a procedure reported in the literature [47,48].

Baking tests were then performed to evaluate the quality of the cupcakes. The dough was re-prepared with yeast, distributed into silicone baking cups (40 ± 1 g), and baked in a convection oven (Unox XF013, Padova, Italy) at 160 °C for 15 min [49].

The images used in the questionnaire were obtained from these experimentally produced samples. The visual characteristics of the products, including color and texture, were directly influenced by the inclusion of insect flour, and no artificial modifications or image processing were applied. The visual stimulus was saved in JPEG format with a resolution of 600 dpi and was displayed identically to all participants within the online survey interface, without resizing by the authors.

**Figure 1:**
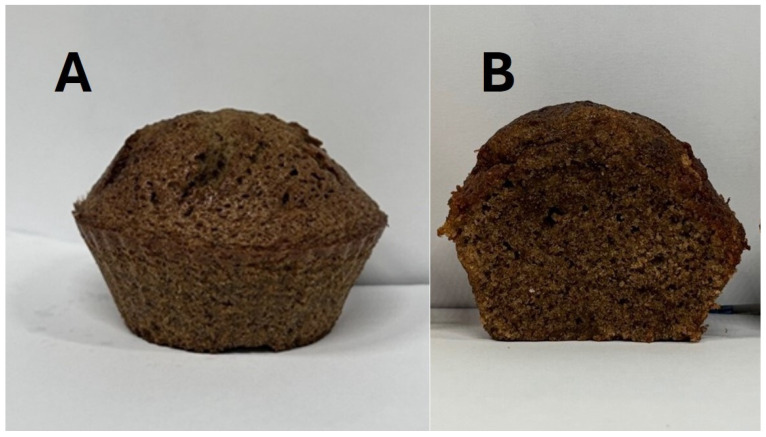
Visual stimuli used in the survey: (**A**) external appearance and (**B**) internal structure of the cupcake prepared with TM flour. The images were presented to respondents for attractiveness evaluation before and after information disclosure.

Although multiple formulations were developed during the product preparation phase, a single representative image corresponding to the formulation containing 10% TM flour was selected as a visual stimulus. Both the external appearance and the internal structure of the product were included in order to provide a more comprehensive visual evaluation. This choice was made to ensure consistency in the visual stimuli and to allow a clearly perceivable visual effect within current regulatory limits (Figure 1).

The use of a single visual stimulus was an intentional choice aimed at maximizing experimental control and isolating the effect of ingredient disclosure on product evaluation. By exposing all respondents to the same image, variability related to product category, presentation, and image characteristics was minimized, allowing a more direct comparison between PRE and POST attractiveness ratings.

### 2.4. Statistical Processing of Data

All statistical analyses and PCA plot were conducted using the open-source statistical software JAMOVI (version 2.6.44.0, The Jamovi project, 2025, Retrieved from https://www.jamovi.org). Additionally, any necessary graphical outputs were created using GraphPad Prism version 8 (GraphPad Software, San Diego, CA, USA). Before each analysis, data were reviewed for completeness, and respondents with missing values relevant to each test were removed.

The appeal ratings collected before (PRE) and after (POST) the disclosure of the insect-based ingredient were treated as paired ordinal variables. Because normality was violated (Shapiro–Wilk test), the difference between PRE and POST ratings was assessed using the Wilcoxon signed-rank test. The associated *p*-value indicates statistical significance (*p* < 0.05), whereas the effect size (r) reflects the magnitude of the difference (0.10–0.29 small, 0.30–0.49 medium, ≥0.50 large).

To evaluate whether the change in attractiveness (Δ = POST − PRE) differed across sociodemographic categories, the normality of the data was examined using the Shapiro–Wilk test. When this assumption was met (*p* > 0.05), one-way ANOVA with Tukey’s HSD post hoc test was applied; otherwise, the non-parametric Kruskal–Wallis test, followed by Dunn’s test with Bonferroni correction, was used for multiple-group comparisons. Additionally, Spearman rank-order correlations were computed to examine monotonic associations between Δ and ordinal predictors (i.e., habitual consumption frequency).

The internal consistency of the attitudinal (ATT) and knowledge-related (KNO) item sets was assessed using Cronbach’s alpha. Given the exploratory nature of the study, Cronbach’s alpha was used as an empirical measure of internal consistency rather than as a criterion for scale construction [50].

The change in attractiveness (Δ) was correlated with attitudinal (ATT) items and with cognitive (KNO) items, using Pearson correlation coefficients, generating two correlation matrices that were visualized through dedicated heatmaps. Although attitudinal and knowledge-related variables were measured using 5-point Likert scales, Pearson correlation coefficients were computed by treating the items as approximately interval variables, in line with common practice for Likert-type scales with five or more categories in exploratory analyses [51,52].

Before PCA, the suitability of the data was assessed using the Kaiser–Meyer–Olkin (KMO) measure of sampling adequacy and Bartlett’s test of sphericity. The overall KMO value indicated good sampling adequacy (KMO = 0.849), and Bartlett’s test was statistically significant (χ^2^ = 5104.317, df = 171, *p* < 0.001), confirming that the correlation matrix was appropriate for PCA.

Principal Component Analysis (PCA) was applied to the ATT and KNO items to explore the underlying structure of participants’ responses and to identify the major latent dimensions. Since all variables were measured on 5-point Likert scales, the analysis was performed on the correlation matrix, after standardizing all items using z-scores, which is the recommended approach for ordinal-approximately-interval data in exploratory PCA. The resulting component scores were visualized in a two-dimensional scatterplot to assess similarities and differences among respondents.

## 3. Results

### 3.1. Survey Participant Characteristics

**Figure 2:**
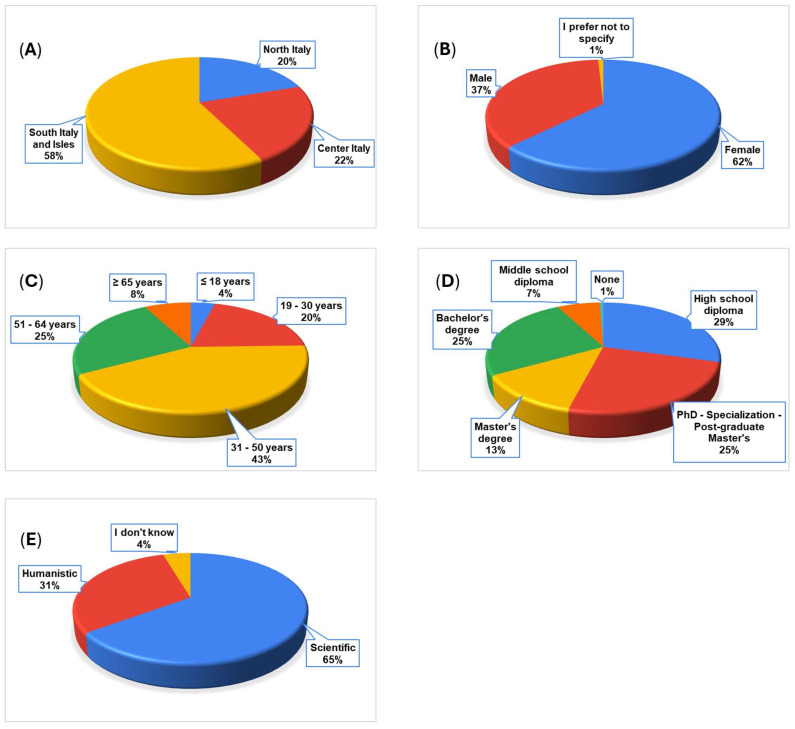
Main characteristics of survey participants: (**A**) origin, (**B**) gender, (**C**) age, (**D**) education level, (**E**) educational background.

Based on the demographic and educational distributions obtained from the survey dataset, the profile of the average respondent is summarized in Figure 2.

The sample (n = 350) consisted predominantly of respondents living in Southern Italy and the Islands (58%) and of female participants (62%). Adults aged 31–50 years were the most represented age group (43%). Educational attainment was generally high, with 63% holding a Bachelor’s, Master’s, or postgraduate degree. Most respondents had a scientific background (65%). Overall, the average participant was a highly educated, middle-aged adult with scientific training.

### 3.2. Consumption Frequency of Bakery Products

**Figure 3:**
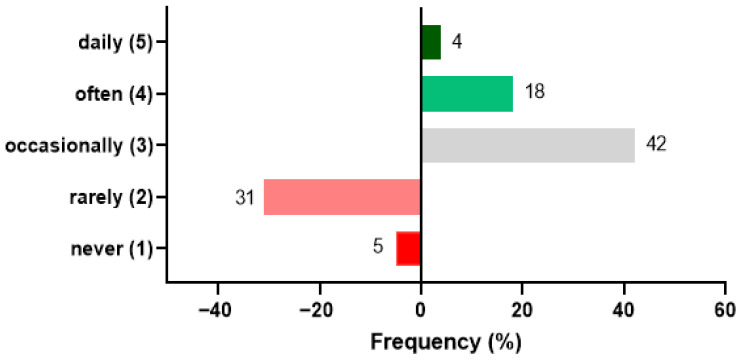
Distribution of the self-reported consumption frequency of soft baked products, displayed as a diverging stacked bar chart based on a 5-point frequency scale (1 = never; 2 = rarely; 3 = occasionally; 4 = often; 5 = daily). Categories 1–2 are plotted on the left of the central axis, whereas categories 3–5 extend to the right.

As shown in Figure 3, most respondents reported consuming soft baked products “occasionally” (42%) or “rarely” (31%), whereas frequent consumption was less common (18%). Extreme categories were minimally represented (5% “never”, 4% “daily”). The mean score (2.85) fell slightly below the midpoint of the scale, indicating moderate familiarity with the product category, albeit without regular consumption.

### 3.3. Effect of Information on Product Attractiveness

**Figure 4:**
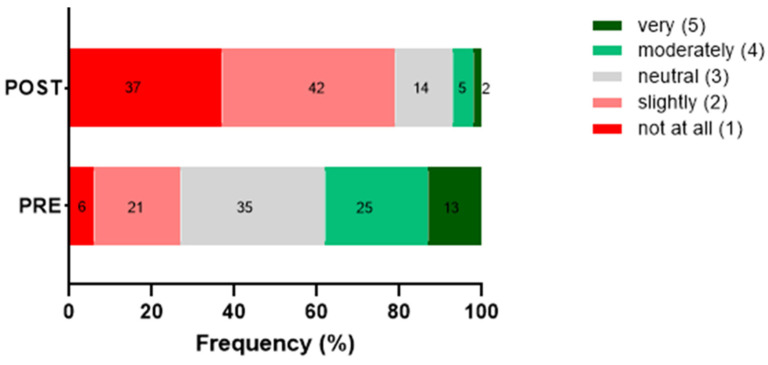
Distribution of appeal ratings for the product before (PRE) and after (POST) the disclosure of the insect-based ingredient. Each bar represents the percentage of responses across the 5-point Likert scale (1 = not at all; 2 = slightly; 3 = neutral; 4 = moderately; 5 = very). Colors correspond to the five rating categories, from low appeal (red tones) to neutral (grey) and higher appeal (green tones).

Participants first rated the attractiveness of the product based solely on the image, and subsequently re-rated it after being informed that it contained 10% TM flour. The distribution of appeal ratings before and after the disclosure of the insect-based ingredient is shown in Figure 4.

Before disclosure (PRE), visual attractiveness was mainly rated as neutral or moderately positive, with 35% and 25% of responses, respectively. After disclosure of the 10% TM flour content (POST), evaluations shifted markedly toward lower scores, with 79% selecting ratings of 1–2. Mean attractiveness decreased from 2.6 (PRE) to 2.0 (POST) (Δ = −0.63; 95% CI: −0.73 to −0.53), demonstrating a pronounced negative impact of the information.

#### 3.3.1. Influence of Sociodemographic Variables on Pre- and Post-Disclosure Evaluations

Differences in product attractiveness across sociodemographic categories were examined by comparing PRE scores (visual evaluation), POST scores (evaluation after disclosure of insect flour), and the change in attractiveness (Δ = POST − PRE). Mean values and standard deviations for each subgroup are reported in Table 3, together with significance groupings obtained from post hoc tests.

Significant differences in PRE scores emerged only for educational background, with humanities-trained participants providing lower initial ratings (2.42 ± 1.04) than those with scientific training (2.73 ± 1.02).

POST scores differed significantly by gender and background: women and humanities-trained respondents rated the product less favorably. The change in attractiveness (Δ = POST − PRE) varied significantly only by gender, with women showing a larger decline than men (−0.74 ± 0.96 vs. −0.44 ± 0.90); this indicates that women not only evaluated more negatively, but also changed more strongly in response to the disclosure. No significant differences were observed across age, geographical area, or education level.

To complement the ANOVA results and assess within-group changes, paired PRE and POST scores were analyzed using the Wilcoxon signed-rank test for each subgroup. Table 4 reports the Wilcoxon *p*-values and the corresponding effect sizes (r), providing insight into the magnitude and significance of change occurring within each subgroup.

Wilcoxon tests confirmed a significant PRE–POST reduction for nearly all subgroups, with medium-to-large effect sizes (r ≈ 0.40–0.60).

The only subgroup that did not show a significant PRE–POST difference was the ≤18 age group, whose evaluations remained essentially stable (*p* = 0.527; r = 0.17). This clarifies the ANOVA finding: although adults show strong within-group decreases, these decreases are of similar magnitude across adult age groups, resulting in non-significant between-group differences.

#### 3.3.2. Influence of Habitual Consumption of Baked Goods on the Change in Product Attractiveness

The effect of habitual consumption frequency on PRE, POST and Δ scores was examined using the Kruskal–Wallis test. Mean values and significance groupings are reported in Table 5.

Habitual consumption affected PRE and Δ, but not POST ratings. Participants who never consumed baked goods provided lower PRE scores than occasional consumers. The decline in attractiveness was smallest among “rarely” consumers and largest among “occasional” consumers, while intermediate groups fell between these extremes.

To complement the between-group analysis, Wilcoxon signed-rank tests were performed within each consumption category to evaluate the effect of information disclosure independently for each group. Wilcoxon tests confirmed significant reductions for “rarely”, “occasionally”, and “often” consumers. Changes in the “never” and “daily” groups did not reach significance, likely due to small sample sizes.

To explore the type and direction of the association between habitual consumption frequency of baked goods and the change in product attractiveness (Δ), a Spearman rank-order correlation was performed. Results showed a small, significant negative correlation (ρ = −0.18), meaning that more frequent consumers were less affected by the disclosure.

Taken together, these findings show that habitual intake of baked products slightly modulates consumer response to the ingredient disclosure. Individuals with more regular exposure to this product category seem to experience a milder penalty in attractiveness, whereas those with limited or occasional consumption tend to display a stronger negative shift.

### 3.4. Consumer Attitudes Towards Insect-Based Food Products

Consumer attitudes were evaluated through eleven items on IBFs. Before examining attitudinal patterns, the internal consistency of the ATT item set was evaluated and showed a Cronbach’s alpha of 0.56, reflecting their intentionally heterogeneous and multidimensional nature, as they were designed to capture distinct affective, cognitive, and behavioral components rather than a unidimensional scale [53]. Accordingly, ATT items were analysed individually rather than as a composite score. Participants indicated their level of agreement using a 5-point Likert scale (Table 1). Each item was associated with a short descriptive label (hereafter referred to as “short label”), as reported in Table 6, to facilitate interpretation of the results.

Overall, responses were predominantly distributed around neutral to moderately positive levels, indicating a general attitude of cautious openness rather than strong acceptance.

Positive perceptions were mainly associated with the potential future diffusion of IBFs and their perceived safety under controlled production conditions, which were generally evaluated at moderate agreement levels. In contrast, concerns about potential health risks tended to remain at low levels of agreement, suggesting limited perceived risk among respondents. At the same time, emotional and behavioral responses appeared more critical. Discomfort associated with consuming IBFs was moderate, while willingness to try or regularly consume such products remained low, with responses clustering around slight and neutral agreement levels. Even when the product was perceived as visually acceptable, the idea of insect content and concerns about taste continued to represent relevant barriers, indicating that cognitive awareness does not necessarily translate into behavioural acceptance.

#### Correlation Analysis Between the Change in Product Appeal and the Attitudinal Items

**Figure 5:**
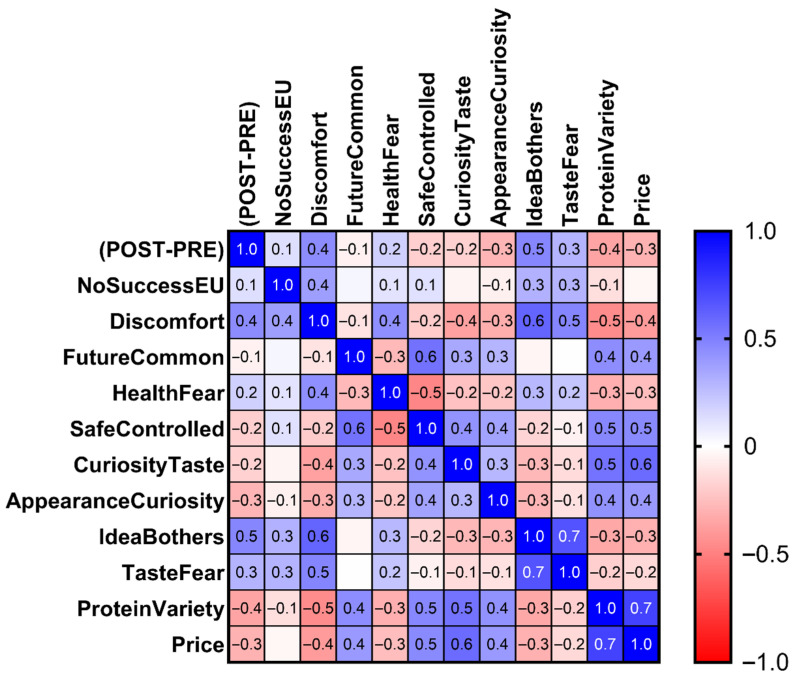
Pearson correlation heatmap between the change in attractiveness (Δ = POST − PRE) and the attitudinal items. Cells display Pearson r values. All coefficients are shown. Blue indicates positive correlations, red indicates negative correlations, and color intensity reflects the magnitude of the coefficient.

The correlation heatmap between the change in attractiveness (Δ = POST − PRE) and the attitudinal items reveals a coherent negative association pattern (Figure 5). Each cell represents the Pearson r between Δ and the corresponding item; all coefficients are shown. Items regarding discomfort, health-related concerns, and aversive reactions (e.g., *Discomfort*, *HealthFear*, *IdeaBothers*, *TasteFear*) show moderate negative correlations with Δ (approximately between −0.3 and −0.6), indicating that respondents expressing stronger negative attitudes tend to experience a greater reduction in product appeal following disclosure. Conversely, items reflecting curiosity or openness toward IBFs (e.g., *CuriosityTaste*, *AppearanceCuriosity*) show weak or slightly positive correlations with Δ, suggesting that exploratory attitudes are associated with a less pronounced decrease in attractiveness.

### 3.5. Consumer Knowledge and Awareness of Insect-Based Foods

Participants’ prior knowledge and awareness of IBFs were assessed through eight items covering regulatory, nutritional, and environmental aspects. The KNO items showed high internal consistency (Cronbach’s alpha = 0.92), supporting their treatment as a coherent set of knowledge indicators in subsequent analyses. Responses were collected using a 5-point Likert scale (Table 1). Each item was associated with a short label, as reported in Table 7.

Respondents showed moderate to high levels of awareness across the different topics. Higher levels of awareness were mainly associated with the versatility of insect flour for food production and the role of insects as alternative protein sources, as well as with the expected increase in global protein demand. Environmental and nutritional aspects were also generally perceived with moderate levels of awareness, indicating a relatively good understanding of the potential benefits associated with IBFs. In contrast, lower levels of awareness were observed for regulatory aspects. In particular, knowledge related to European authorizations, the possibility of consuming whole insects, and national limits on insect flour inclusion showed comparatively lower levels of awareness.

#### Correlation Analysis Between the Change in Product Appeal and the Cognitive Items

**Figure 6:**
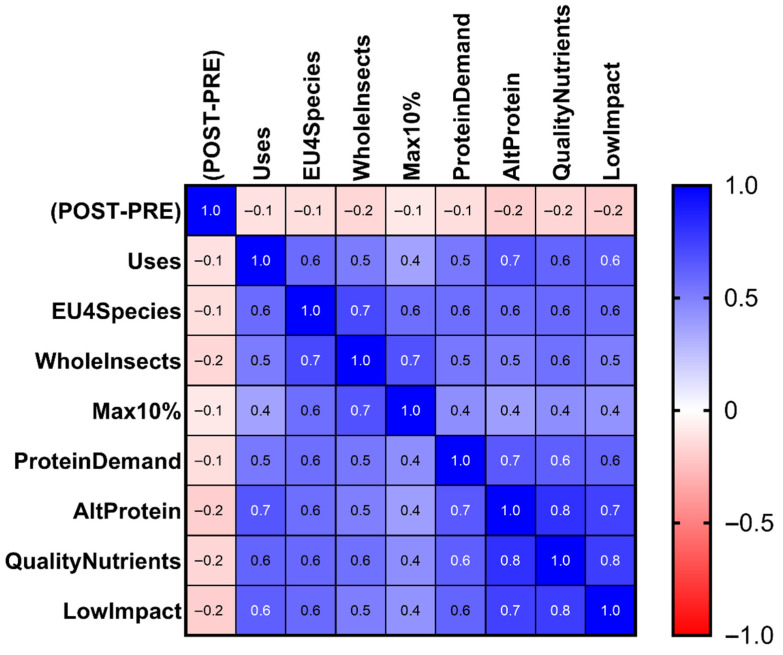
Pearson correlation heatmap between the change in attractiveness (Δ = POST − PRE) and the knowledge-related items. Cells display Pearson r values. All coefficients are shown. Blue indicates positive correlations, red indicates negative correlations, and color intensity reflects the magnitude of the coefficient.

The correlation heatmap examining Δ in relation to the knowledge-related items (Figure 6) shows a distinct and consistently positive profile, contrasting with the pattern observed for attitudinal variables in Figure 5. KNO items showed consistently positive correlations with Δ (approximately +0.3 to +0.7).

This homogeneity suggests that respondents with greater informational awareness or more developed cognitive frameworks regarding IBFs tend to exhibit a smaller reduction in attractiveness after disclosure. Unlike the attitudinal domain, the KNO heatmap does not display divergent clusters; instead, it reflects a uniform cognitive orientation in which knowledge and perceived understanding play a protective role against the decrease in appeal.

In summary, while attitudinal variables relate to Δ in a valence-dependent direction (more negative attitudes → larger drop), cognitive variables show a mitigating effect, supporting a more stable evaluation after disclosure.

### 3.6. Principal Component Analysis (PCA) of Attitudinal and Cognitive Items

A Principal Component Analysis (PCA) was conducted on ATT and KNO items to identify the latent psychological dimensions underlying consumer perceptions of IBFs. All items, measured on 5-point Likert scales, were standardized using z-scores before analysis. Respondents with missing values in any of the 19 items were removed listwise to ensure stable component extraction and consistent interpretation of the factor structure.

The PCA yielded a two-component solution, selected based on eigenvalues greater than 1 and inspection of the scree plot. Together, the two components accounted for a substantial proportion of the total variance, revealing a clear underlying structure in the combined attitudinal and knowledge-related responses.

As shown in Supplementary Material, Section S2, Table S1, the first principal component (PC1), explaining 61.6% of the total variance, represents the dominant attitudinal dimension differentiating respondents. The interpretation of this component is supported by the pattern of factor loadings, with items reflecting curiosity, perceived safety under controlled production, and openness toward IBFs showing positive loadings, whereas items related to discomfort, perceived health risks, and rejection showed negative loadings. PC1, therefore, captures a continuum ranging from emotional aversion to acceptance and constitutes the primary axis separating consumer profiles.

The second component (PC2), accounting for 33.3% of the variance, reflects secondary differences mainly related to prior knowledge and informational awareness. This interpretation is also supported by the loading structure, as knowledge-related items showed consistent contributions to this component, differentiating respondents who were more familiar with regulatory, nutritional, and environmental aspects of IBFs with limited prior exposure. PC2 contributes to within-group variability but plays a less prominent role in distinguishing consumer clusters.

**Figure 7:**
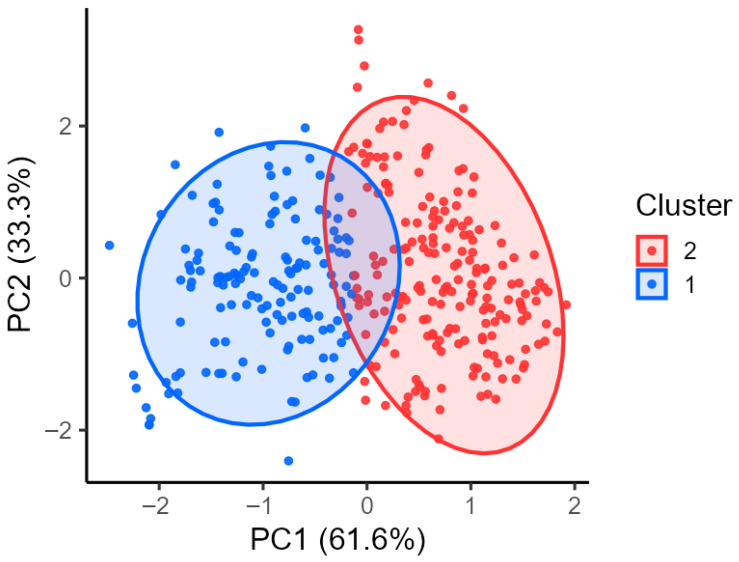
Each point represents a participant, and the ellipses—derived from the covariance structure of the PCA scores—illustrate the multivariate dispersion of two psychological clusters identified via k-means clustering (k = 2). The blue ellipse corresponds to individuals scoring higher on PC1, indicating greater openness and acceptance, whereas the red ellipse includes respondents characterized by lower PC1 scores, reflecting more negative or skeptical attitudes. Although some overlap between the clusters is observed, their centroids are clearly separated along the PC1 axis, confirming that attitudinal acceptance is the main dimension differentiating consumers, while PC2 captures secondary variation related to knowledge and awareness.

To visualize the distribution of respondents across these latent dimensions, PCA scores for PC1 and PC2 were plotted in a two-dimensional ordination space (Figure 7).

Each point represents a participant, and the ellipses—derived from the covariance structure of the PCA scores—illustrate the multivariate dispersion of two psychological clusters identified via k-means clustering (k = 2). The ellipses are intended to represent within-cluster variability rather than statistical boundaries or tests of separation. The blue ellipse corresponds to individuals scoring higher on PC1, indicating greater openness and acceptance, whereas the red ellipse includes respondents characterized by lower PC1 scores, reflecting more negative or skeptical attitudes. Although some overlap between the clusters is observed, their centroids are clearly separated along the PC1 axis, confirming that attitudinal acceptance is the main dimension differentiating consumers, while PC2 captures secondary variation related to knowledge and awareness.

The PCA configuration is consistent with the behavioral patterns observed in the PRE–POST ratings (Section 3.3). Individuals scoring high on PC1—located predominantly within the blue ellipse—tended to provide higher POST values and smaller Δ (POST−PRE) declines, reflecting their more favorable or rational orientation toward IBFs. Conversely, participants characterized by higher PC2 scores and lower PC1 scores—primarily within the red ellipse—exhibited substantially lower POST values and larger negative Δ, indicating that emotional aversion strongly amplifies the detrimental effect of ingredient disclosure. Thus, PCA offers a coherent psychological explanation for the observed heterogeneity in PRE and POST responses: attitudinal openness mitigates the negative impact of information, whereas visceral aversion intensifies it.

## 4. Discussion

The sociodemographic profile of the sample examined in this study partially aligns with national population patterns and reflects characteristics commonly associated with online survey-based data collection.

In terms of gender, females were overrepresented in the sample (62%) compared to the Italian population, which is approximately evenly distributed (51% female) [49]. This pattern is consistent with previous Italian studies reporting a higher participation of women in voluntary online surveys [44,54,55]. Globally, a large body of literature supports the observation that women tend to participate in surveys more frequently and more readily than men, demonstrating a substantial and systematic gender difference in online survey engagement [56,57].

The age distribution was mainly concentrated in the 31–50 group (43%), one of the most populous adult segments in Italy. Conversely, older individuals (≥65 years), who represent approximately 24% of the national population [49], were underrepresented, likely due to their lower engagement with digital survey tools. A more pronounced deviation was observed in the geographical distribution. Respondents from Southern Italy and the Islands accounted for 58% of the sample, whereas these regions represent roughly 34–36% of the national population [49]. This imbalance likely reflects the dissemination channels used for survey distribution rather than systematic sampling bias.

The sample was also characterized by a relatively high education level. Participants holding tertiary education degrees (Bachelor’s, Master’s, or postgraduate qualifications) exceeded national estimates, where individuals with such qualifications represent approximately 20–25% of the adult population [49]. Similar deviations from the national population structure have also been reported in studies on Italian consumers [58].

In addition, the predominance of respondents with a scientific background (65%) suggests a sample with comparatively high familiarity with sustainability-related and scientific topics.

Overall, while the sample cannot be considered fully representative of the Italian population, it reflects a specific and relevant segment of consumers—namely, highly educated and cognitively engaged individuals—who provide valuable insights for studying perceptions and acceptance of novel food products.

Building upon this context, the findings of this study contribute to the growing body of literature investigating the factors that shape Western consumers’ acceptance of IBFs. The results clearly show that visual familiarity supports initial neutrality or partial acceptance; however, information disclosure regarding insect ingredients triggers a strong negative shift in perceptions—an effect widely documented across Western populations. This shift aligns with previous research showing that disgust sensitivity, food neophobia, and cultural distance from entomophagy are key predictors of rejection of IBFs, even when the product’s sensory properties are acceptable, and the insect’s ingredient is hidden [27,59,60,61]. While this decrease is consistent with previous literature, the present study provides novel insights by isolating the visual component of food evaluation and quantitatively linking the magnitude of the PRE–POST shift to both attitudinal and knowledge-related factors. In particular, the integration of correlation analysis and PCA highlights how affective responses and cognitive awareness jointly shape consumer reactions, offering a more structured interpretation of the mechanisms underlying acceptance of insect-based foods. The pre–post design adopted in this study reinforces this interpretation. Before disclosure, participants evaluated the cupcake as moderately attractive, indicating that processed IBFs without visible insect parts can benefit from perceptual familiarity, as previously observed in several studies comparing whole-insect versus flour-based formats [20,61]. However, once informed of the presence of TM flour, attractiveness scores declined sharply across almost all subgroups, confirming that psychological and affective barriers override visual cues. Similar results were reported by Cicatiello et al. (2020) [62], who observed significant decreases in liking after ingredient disclosure, particularly when insects were visible, and by Arena et al. (2020), who found that informational cues enhance neophobic responses [63].

Attitudinal patterns observed in this study further support the central role of affective dispositions in shaping acceptance. In line with previous literature, the emotional responses discussed in the present study may reflect different underlying mechanisms contributing to consumer rejection of insect-based foods. Disgust can be interpreted as a primary and immediate affective reaction linked to food rejection, whereas concerns related to health or safety may reflect more cognitive forms of risk perception. In addition, the idea of consuming insect-based foods may evoke symbolic or moral forms of aversion associated with cultural norms, food identity, and deeply ingrained representations of what is considered acceptable as food. Although the present study did not explicitly distinguish between these emotional components, the observed response patterns suggest that multiple affective pathways may jointly contribute to the marked decrease in attractiveness following ingredient disclosure.

Items measuring discomfort, taste-related concerns, or psychological aversion showed the strongest negative association with post-disclosure attractiveness ratings, consistent with meta-analytic evidence indicating that disgust and food neophobia are the most powerful predictors of rejection [28]. Studies from Germany and Italy converge on this point: Lammers et al. (2019) [64] found food disgust to be the primary determinant of willingness to consume insects in the German population, while Serpico et al. (2021) [45] documented persistent “insect phobia” despite growing exposure and information. Similarly, Verbeke (2015) [27] identified low food neophobia and sensation-seeking traits as key factors characterizing the minority of early adopters.

Interestingly, the present study also confirms the role of cognitive knowledge. Moreover, it is important to distinguish between objective knowledge (i.e., factual awareness of regulatory, nutritional, and environmental aspects) and subjective knowledge (i.e., perceived understanding or familiarity). In the present study, knowledge was assessed through self-reported awareness indicators, which reflect respondents’ cognitive orientation toward insect-based foods rather than verified factual accuracy. The results suggest that increased awareness alone may not be sufficient to overcome negative emotional responses, highlighting the need to jointly consider cognitive and affective dimensions when explaining consumer acceptance.

Although awareness of nutritional and environmental benefits did not increase willingness to try the cupcake, participants with higher knowledge scores exhibited a less pronounced decline in attractiveness following disclosure. This pattern mirrors findings by Rehman and Ogrinc (2024) [61] in Slovenia, where higher informational awareness—particularly sustainability-related—was associated with greater openness to insect-based products. However, alternative explanations should also be considered. The observed decrease in attractiveness may be partially influenced by survey-related factors, such as the hypothetical nature of the evaluation and the absence of real sensory experience. In addition, the use of a single visual stimulus may have amplified stimulus-specific reactions, potentially affecting the magnitude of the PRE–POST shift independently of broader perceptions of insect-based foods.

Sociodemographic differences observed in this study align with well-documented patterns in the literature. Female participants expressed more negative post-disclosure evaluations and sharper declines in attractiveness, consistent with international evidence indicating that women tend to exhibit higher disgust sensitivity and stronger neophobic responses [28,45,61]. In this context, the stronger negative shift observed among female participants following information disclosure may be interpreted as the activation of affective and risk-avoidance mechanisms, which override initial visual acceptance, suggesting that emotional responses play a dominant role in shaping female consumers’ reactions to insect-based foods. The lower acceptance observed among participants with a humanistic background may reflect reduced exposure to scientific content related to sustainability, innovation, or alternative protein systems. Although the literature does not directly compare humanistic versus scientific educational backgrounds, several studies demonstrate that greater scientific literacy, familiarity with environmental issues, and exposure to science-based information are associated with more favorable attitudes toward IBFs. In particular, education level and exposure to scientific content influence food neophobia and acceptance [58]. It is therefore plausible that lower exposure to scientific and sustainability-related knowledge frameworks contributes to a greater reliance on affective or intuitive judgments, which are widely recognized as major drivers of rejection in Western contexts. By contrast, age and education showed weaker or inconsistent associations, a finding fully aligned with large-scale analyses indicating that sociodemographic traits (including age, gender, education, and residence) are generally poor predictors of willingness to consume insects. Meta-analytic evidence demonstrates that these variables carry minimal explanatory power compared with affective constructs such as disgust, neophobia, prior experience, and emotional responses [28,59,65,66]. In addition, the predominance of respondents with a scientific background may have influenced the overall findings, as this group is generally characterized by higher familiarity with sustainability and novel food concepts. This may have contributed to relatively high baseline awareness and a more cognitively structured evaluation of IBFs compared to the general population. However, despite this potential bias toward a more informed sample, the observed decrease in attractiveness following information disclosure remained substantial, indicating that the negative impact of insect ingredient awareness is robust even among highly educated and scientifically oriented consumers.

The PCA results of this study provide additional interpretative depth by identifying two latent dimensions: an attitudinal continuum ranging from aversion to acceptance, and a cognitive dimension related to informational awareness. This structure closely parallels theoretical frameworks proposed in the literature, where psychological dispositions—particularly disgust—form the core barrier, while cognitive evaluations (e.g., sustainability, nutrition) contribute to moderating but not reversing aversive reactions. Several studies and reviews similarly argue that emotional resistance dominates consumer responses, and that acceptance pathways require strategies targeting both affective and cognitive components [28,59,65,66]. Overall, these findings confirm the longstanding challenge documented across Western societies: the persistent gap between conceptual acceptance (e.g., sustainability awareness) and behavioral intention to consume IBFs. Despite increasing knowledge about nutritional and environmental benefits, the psychological burden of disgust remains difficult to overcome, as highlighted in systematic reviews emphasizing the predominance of emotional, cultural, and symbolic dimensions in shaping acceptance [66,67,68,69].

This study also presents several limitations. The snowball sampling approach led to overrepresentation of women, highly educated individuals, and respondents from Southern Italy. While these patterns are typical of online surveys, they limit the generalizability of the findings. A further limitation is related to the visual design of the experiment. The use of a single image as a visual stimulus was intentionally adopted to ensure strict control over visual attributes and to isolate the effect of information disclosure on product evaluation. However, relying on a single visual representation may reduce the robustness of the findings, as participants’ responses could partly reflect stimulus-specific features rather than generalized reactions to insect-based foods across different product formats, visual designs, or presentation contexts. Moreover, the exclusive focus on visual evaluation, while useful for isolating perceptual mechanisms, does not capture actual sensory experiences such as taste, texture, or odor, which are crucial determinants of food acceptance. In addition, only one product category and one insect-flour inclusion level were considered. Future research should therefore adopt multi-sensory and experimental tasting designs, include repeated exposure conditions, and test multiple product typologies and visual presentations to assess the stability of the observed effects under more realistic consumption scenarios. Such directions are also consistent with evidence from studies conducted in different cultural and national contexts, which suggest that familiarity and repeated exposure may contribute to reducing affective and neophobic barriers toward insect-based foods [28].

Finally, the fixed pre–post structure of the questionnaire implies that post-disclosure evaluations may have been influenced by anchoring to the initial visual assessment. However, this effect is intrinsic to the pre–post design adopted in the study and is conceptually aligned with the research objective, which was to examine how information disclosure reshapes previously formed visual impressions rather than eliciting independent evaluations.

From an applied perspective, these findings suggest that the negative impact of ingredient disclosure cannot be mitigated by information alone, as increased knowledge was associated with only a partial attenuation of the PRE–POST decline. This indicates that effective strategies should combine cognitive and affective approaches, integrating consumer education with product design elements that enhance familiarity and reduce perceptual distance. In this context, gradual exposure and the use of familiar food formats may represent more effective pathways for improving acceptance than information-based interventions alone.

This study reinforces the central role of emotional and cognitive factors in shaping consumer acceptance of IBFs. While visual familiarity supports initial openness, information disclosure activates psychological barriers that substantially reduce acceptance. Overcoming these barriers will require strategies that simultaneously address perceptual familiarity, emotional resistance, and knowledge gaps. Insights from international research suggest that successful integration of IBFs into Western markets depends not only on product innovation and sensory optimization, but also on culturally sensitive communication and progressive exposure approaches.

## 5. Conclusions

This study demonstrates that, although visually familiar insect-based products can elicit neutral to moderately positive initial responses, the disclosure of insect-derived ingredients remains a critical barrier to consumer acceptance. The marked decline in attractiveness observed after revealing the presence of TM flour highlights the predominance of psychological and emotional responses—particularly disgust and food neophobia—in shaping consumer evaluations, in line with the broader literature on Western consumer behavior toward IBFs.

At the same time, cognitive factors, such as awareness of nutritional and environmental benefits, play a moderating—yet not decisive—role, as better-informed individuals exhibited less pronounced negative shifts. However, the persistence of attitudinal resistance, despite generally high levels of awareness, confirms that information alone is insufficient to drive behavioral adoption.

Overall, these findings underscore the need for multifaceted strategies that combine product familiarization, targeted communication, and consumer exposure in real sensory contexts. Future research should integrate visual and sensory evaluation, investigate the effects of repeated exposure, and assess communication approaches capable of reducing emotional barriers. Such efforts will be essential to support the gradual integration of IBFs into Western diets and to unlock their potential contribution to more sustainable food systems.

## Figures and Tables

**Table 1:** Overview of questionnaire sections and variables, corresponding labels, data types (Categorial or Likert), and measurement scales used in the survey.

Section	Variable	Label	Type ^1^	Scale
Sociodemographic data	Origin	-	C	North Italy
				Centre Italy
				South Italy + Isles
			
	Gender	-	C	Female
				Male
				Prefer not specify
			
	Age	-	C	≤18 years
				19–30 years
				31–50 years
				51–64 years
				≥65 years
			
	Education level	-	C	Advanced Degrees (*PhD–Specialization–Post-graduate Master’s*)
				Master’s degree
				Bachelor’s degree
				High school diploma
				Middle school diploma
				None
			
	Educational background	-	C	Scientific
				Humanistic
				Don’t Know
				
Consumption habits	Consumption	-	F	1 = never
				2 = rarely
				3 = occasionally
				4 = often
				5 = daily
				
Visual Evaluation	Attractiveness before disclosure of insect ingredient	PRE	L	1 = not at all
				2 = slightly
				3 = neutral
				4 = moderately
				5 = very
			
	Attractiveness after disclosure of insect ingredient	POST	L	1 = not at all
				2 = slightly
				3 = neutral
				4 = moderately
				5 = very
				
Attitudes	11 items on perception, risk, and acceptance of insect-based foods	ATT	L	1 = not at all
				2 = slightly
				3 = neutral
				4 = moderately
				5 = very
				
Knowledge	8 items on regulatory, nutritional, and environmental aspects	KNO	L	1 = not at all
				2 = slightly
				3 = neutral
				4 = moderately
				5 = very

^1^ C = categorial; F = frequency; L = likert.

**Table 2:** Cupcakes recipe. C: standard recipe; CO: cupcake formulation with sunflower oil; CO5%TM: CO containing 5% *w*/*w* TM flour; CO10%TM: CO containing 10% *w*/*w* TM flour.

Component	C % *w*/*w*	CO % *w*/*w*	CO5%TM % *w*/*w*	CO10%TM % *w*/*w*
Flour 00	22.4	24.0	19.0	14.4
TM flour	-	-	5.0	9.6
Milk	10	10.7	10.7	10.7
Butter	22.4	-	-	-
Sunflower oil	-	16.8	16.8	16.8
Sugar	22.4	24.0	24.0	24.0
Egg	22.4	24.0	24.0	24.0
Yeast	0.4	0.5	0.5	0.5

**Table 3:** Product attractiveness (PRE, POST) and change in attractiveness (Δ) across sociodemographic groups.

Variable	Groups	Question PRE	Question POST	Δ (POST − PRE)
Origin	North Italy	2.81 ± 1.03	2.26 ± 1.13	−0.56 ± 0.93
	Centre Italy	2.58 ± 1.10	2.01 ± 1.04	−0.56 ± 1.06
	South Italy + Isles	2.59 ± 1.03	1.92 ± 1.03	−0.68 ± 0.92
				
Gender	Female	2.61 ± 1.03	1.88 ± 1.02 ^a^	−0.74 ± 0.96 ^b^
	Male	2.66 ± 1.05	2.22 ± 1.06 ^b^	−0.44 ± 0.90 ^a^
	Prefer not specify	3.33 ± 2.08	2.33 ± 2.31 ^ab^	−1.00 ± 1.73 ^ab^
				
Age	≤18 years	2.71 ± 1.07	2.57 ± 1.02	−0.14 ± 0.86
	19–30 years	2.64 ± 1.00	2.10 ± 0.98	−0.54 ± 1.01
	31–50 years	2.62 ± 1.05	1.99 ± 1.07	−0.63 ± 0.90
	51–64 years	2.74 ± 1.04	1.94 ± 1.08	−0.80 ± 1.03
	≥65 years	2.30 ± 1.14	1.74 ± 1.06	−0.56 ± 0.85
				
Education level	Advanced Degrees	2.67 ± 1.03	2.16 ± 1.06	−0.51 ± 0.98
	Master’s degree	2.73 ± 1.05	2.06 ± 1.11	−0.67 ± 0.93
	Bachelor’s degree	2.71 ± 1.08	2.09 ± 1.08	−0.62 ± 1.01
	High school diploma	2.49 ± 1.05	1.78 ± 0.96	−0.71 ± 0.94
	Middle school diploma	2.64 ± 0.95	2.00 ± 1.08	−0.64 ± 0.95
	None	2.50 ± 2.12	3.00 ± 1.41	+0.50 ± 0.71
				
Educational background	Scientific	2.73 ± 1.02 ^a^	2.13 ± 1.06 ^a^	−0.59 ± 0.96
	Humanistic	2.42 ± 1.04 ^b^	1.71 ± 0.99 ^b^	−0.71 ± 0.92
	Don’t Know	2.75 ± 1.34 ^ab^	2.19 ± 1.17 ^ab^	−0.56 ± 1.15

Values are mean ± SD. Different letters within each column indicate significant differences, *p* < 0.05.

**Table 4:** Wilcoxon Signed-Rank Test Results (PRE vs. POST).

Variable	Groups	Wilcoxon *p*-Value	Effect Size r ^2^
Origin	North Italy	2.02 × 10^−5^	0.44
	Centre Italy	3.21 × 10^−5^	0.40
	South Italy + Isles	5.85 × 10^−17^	0.42
			
Gender	Female	3.81 × 10^−19^	0.59
	Male	8.05 × 10^−7^	0.45
	Prefer not specify	0.317	0.37
			
Age	≤18 years	**0.527** ^1^	0.17
	19–30 years	7.44 × 10^−5^	0.47
	31–50 years	2.18 × 10^−12^	0.58
	51–64 years	2.03 × 10^−8^	0.60
	≥65 years	0.00177	0.60
			
Education level	Advanced Degrees	2.31 × 10^−5^	0.34
	Master’s degree	1.74 × 10^−8^	0.45
	Bachelor’s degree	0.00057	0.35
	High school diploma	1.06 × 10^−9^	0.47
	Middle school diploma	0.00426	0.44
	None	0.317	0.37
			
Educational background	Scientific	3.50 × 10^−15^	0.49
	Humanistic	1.75 × 10^−10^	0.43
	Don’t Know	0.0528	0.33

^1^ Bold *p*-values mark the only subgroup showing no significant PRE–POST difference (*p* ≥ 0.05). ^2^ Effect-size column (r) reflects conventional magnitude ranges: small (0.10–0.29), medium (0.30–0.49), large (≥0.50).

**Table 5:** Product attractiveness (PRE, POST) and change in attractiveness (Δ) across habitual consumption frequency.

Frequency	PRE (Mean ± SD)	POST (Mean ± SD)	Δ (Mean ± SD)
1 = Never	2.06 ± 1.09 ^a^	1.47 ± 0.71	−0.59 ± 1.12 ^ab^
2 = Rarely	2.47 ± 1.05 ^ab^	2.11 ± 1.07	−0.36 ± 0.82 ^a^
3 = Occasionally	2.79 ± 1.03 ^b^	1.97 ± 1.06	−0.82 ± 1.00 ^b^
4 = Often	2.68 ± 0.99 ^ab^	2.03 ± 1.10	−0.64 ± 0.85 ^ab^
5 = Daily	2.79 ± 1.05 ^ab^	2.07 ± 0.99	−0.71 ± 1.20 ^ab^

Values are mean ± SD. Different letters within each column indicate significant differences, *p* < 0.05.

**Table 6:** Mean agreement scores for items related to consumer attitudes towards IBFs, expressed on a 5-point Likert scale (1 = not at all; 2 = slightly; 3 = neutral; 4 = moderately; 5 = very).

Item	Short Label	Mean
It is unlikely that insect-based products will be successful in Europe	NoSuccessEU	3.16 ± 1.17
I would feel uncomfortable consuming food containing insect flour	Discomfort	3.09 ± 1.47
I believe that insect-based foods may become common in the future	FutureCommon	3.30 ± 1.26
I am concerned that consuming insect-based foods may be harmful to health	HealthFear	2.07 ± 1.19
I believe that insect-based foods can be safe if produced under controlled conditions	SafeControlled	3.56 ± 1.34
I would taste this product out of curiosity	CuriosityTaste	2.24 ± 1.32
My curiosity to try a product strongly depends on its appearance	AppearenceCuriosity	2.28 ± 1.31
Even if the appearance is appealing, the idea that it contains insect flour would prevent me from tasting it	IdeaBothers	2.43 ± 1.46
Even if the appearance is appealing, I am concerned that I might not like its taste	TasteFear	2.22 ± 1.30
I would consume it to diversify my protein sources	ProteinVariety	2.22 ± 1.28
I would try it if the price were lower	Price	2.20 ± 1.31

**Table 7:** Mean scores for items related to consumer knowledge and awareness of IBFs, based on a 5-point Likert scale (1 = not at all; 2 = slightly; 3 = neutral; 4 = moderately; 5 = very).

Item	Short Label	Mean
Insect flour can be used to produce a variety of food products (e.g., pasta, crackers, sweet and savory snacks)	Uses	4.00 ± 1.23
The European Commission has authorized the use of four insect species in powdered form	EU4Species	3.37 ± 1.31
The European Commission allows the consumption of whole insects (if dried or frozen) belonging to authorized species	WholeInsects	2.95 ± 1.27
National Regulation states the insect flour must not exceed 10% of the total ingredients	Max10%	2.71 ± 1.19
Global protein demand will increase in the coming years due to population growth	ProteinDemand	3.67 ± 1.32
Insects provide an alternative protein source to conventional livestock (e.g., cattle, sheep, poultry, fish)	AltProtein	3.90 ± 1.34
Insects contain high-quality proteins, essential amino acids, and important nutrients such as vitamins and minerals	QualityNutrients	3.57 ± 1.35
Insect farming results in lower greenhouse gas emissions, lower water and land use compared to conventional livestock farming	LowImpact	3.61 ± 1.38

## Data Availability

The original contributions presented in this study are included in the article/supplementary material. Further inquiries can be directed to the corresponding author.

## Supplementary Materials

The following supporting information can be downloaded at: https://www.mdpi.com/article/10.3390/foods15101703/s1, Section S1: Questionnaire; Section S2: Table S1. Component loadings for the 19 attitudinal (ATT) and knowledge-related (KNO) items included in the Principal Component Analysis (PCA), as described in Section 3.6. Loadings indicate the strength and direction of the association between each item and the two extracted components (PC1 and PC2). Higher absolute values represent stronger contributions to the underlying latent dimensions.

## Abbreviations

The following abbreviations are used in this manuscript:

ANOVA	Analysis of Variance
ATT	Attitudinal
C	Control cupcake formulation
CO	Cupcake with sunflower oil
CO5%TM	Cupcake with 5% *Tenebrio molitor* flour
CO10%TM	Cupcake with 10% *Tenebrio molitor* flour
EFSA	European Food Safety Authority
ENEA	Italian National Agency for New Technologies, Energy and Sustainable Economic Development
EU	European Union
HSD	Tukey’s Honestly Significant Difference
IBF	Insect-Based Food
IPIFF	International Platform of Insects for Food and Feed
ISTAT	Italian National Institute of Statistics
KNO	Knowledge-related
PC1	First Principal Component
PC2	Second Principal Component
PCA	Principal Component Analysis
POST	Attractiveness rating after disclosure
PRE	Attractiveness rating before disclosure
SD	Standard Deviation
TM	*Tenebrio molitor*
UNICAL	University of Calabria

## References

[1] Gu D., Andreev K., Dupre E.M. (2021). Major Trends in Population Growth Around the World. China CDC Wkly..

[2] Taseska T., Yu W., Wilsey M.K., Cox C.P., Meng Z., Ngarnim S.S., Müller A.M. (2023). Analysis of the Scale of Global Human Needs and Opportunities for Sustainable Catalytic Technologies. Top. Catal..

[3] Leridon H. (2020). Population Mondiale: Vers Une Explosion Ou Une Implosion?. Popul. Soc..

[4] Food and Agriculture Organization (2025). The State of Food Security and Nutrition in the World.

[5] Lisboa H.M., Nascimento A., Arruda A., Sarinho A., Lima J., Batista L., Dantas M.F., Andrade R. (2024). Unlocking the Potential of Insect-Based Proteins: Sustainable Solutions for Global Food Security and Nutrition. Foods.

[6] Leip A., Billen G., Garnier J., Grizzetti B., Lassaletta L., Reis S., Simpson D., Sutton M.A., de Vries W., Weiss F. (2015). Impacts of European Livestock Production: Nitrogen, Sulphur, Phosphorus and Greenhouse Gas Emissions, Land-Use, Water Eutrophication and Biodiversity. Environ. Res. Lett..

[7] Halloran A., Halloran A., Flore R., Paul V., Roos N. (2018). Edible Insects in Sustainable Food Systems.

[8] van Huis A., Rumpold B.A., van der Fels-Klerx H.J., Tomberlin J.K. (2021). Advancing Edible Insects as Food and Feed in a Circular Economy. J. Insects Food Feed.

[9] van Huis A., Gasco L. (2023). Insects as Feed for Livestock Production. Science.

[10] Churchward-Venne T.A., Pinckaers P.J.M., van Loon J.J.A., van Loon L.J.C. (2017). Consideration of Insects as a Source of Dietary Protein for Human Consumption. Nutr. Rev..

[11] Belluco S., Losasso C., Maggioletti M., Alonzi C.C., Paoletti M.G., Ricci A. (2013). Edible Insects in a Food Safety and Nutritional Perspective: A Critical Review. Compr. Rev. Food Sci. Food Saf..

[12] Imathiu S. (2020). Benefits and Food Safety Concerns Associated with Consumption of Edible Insects. NFS J..

[13] Liu A.-J., Li J., Gómez M.I. (2019). Factors Influencing Consumption of Edible Insects for Chinese Consumers. Insects.

[14] Nervo C., Ricci M., Torri L. (2024). Understanding Consumers Attitude towards Insects as Food: Influence of Insect Species on Liking, Emotions, Sensory Perception and Food Pairing. Food Res. Int..

[15] Orkusz A., Orkusz M. (2024). Edible Insects in Slavic Culture: Between Tradition and Disgust. Insects.

[16] European Commission (2022). Commission Implementing Regulation (EU) 2022/169 of 8 February 2022 Authorising the Placing on the Market of Frozen, Dried and Powder Forms of Yellow Mealworm (Tenebrio molitor Larva) as a Novel Food Under Regulation (EU) 2015/2283 of the European Parliament and of the Council, and Amending Commission Implementing Regulation (EU) 2017/2470 (Text with EEA Relevance).

[17] European Commission (2021). Commission Implementing Regulation (EU) 2021/1975 of 12 November 2021 Authorising the Placing on the Market of Frozen, Dried and Powder Forms of Locusta migratoria as a Novel Food Under Regulation (EU) 2015/2283 of the European Parliament and of the Council and Amending Commission Implementing Regulation (EU) 2017/2470.

[18] European Commission (2022). Commission Implementing Regulation (EU) 2022/188 of 10 February 2022 Authorising the Placing on the Market of Frozen, Dried and Powder Forms of Acheta domesticus as a Novel Food Under Regulation (EU) 2015/2283 of the European Parliament and of the Council and Amending Commission Implementing Regulation (EU) 2017/2470.

[19] Téllez-Morales J.A., Hernández-Santos B., Navarro-Cortez R.O., Rodríguez-Miranda J. (2022). Impact of the Addition of Cricket Flour (Sphenarium purpurascens) on the Physicochemical Properties, Optimization and Extrusion Conditions of Extruded Nixtamalized Corn Flour. Appl. Food Res..

[20] International Platform of Insects for Food and Feed (IPIFF) EU Consumer Acceptance of Edible Insects: Survey Report. https://ipiff.org/eu-consumer-acceptance-of-edible-insects-survey-report/.

[21] de Koning W., Dean D., Vriesekoop F., Aguiar L.K., Anderson M., Mongondry P., Oppong-Gyamfi M., Urbano B., Luciano C.A.G., Jiang B. (2020). Drivers and Inhibitors in the Acceptance of Meat Alternatives: The Case of Plant and Insect-Based Proteins. Foods.

[22] Bukchin-Peles S. (2024). Shaping Attitudes toward Sustainable Insect-Based Diets: The Role of Hope. Future Foods.

[23] Omuse E.R., Tonnang H.E.Z., Yusuf A.A., Machekano H., Egonyu J.P., Kimathi E., Mohamed S.F., Kassie M., Subramanian S., Onditi J. (2024). The Global Atlas of Edible Insects: Analysis of Diversity and Commonality Contributing to Food Systems and Sustainability. Sci. Rep..

[24] Tian H., Chen J. (2025). Association of Food Neophobia and Food Disgust with the Willingness, Benefits, and Risks of Insect Food Consumption among Chinese University Students. Front. Nutr..

[25] Birch L.L., McPhee L., Shoba B.C., Pirok E., Steinberg L. (1987). What Kind of Exposure Reduces Children’s Food Neophobia?. Appetite.

[26] Houston-Price C., Butler L., Shiba P. (2009). Visual Exposure Impacts on Toddlers’ Willingness to Taste Fruits and Vegetables. Appetite.

[27] Verbeke W. (2015). Profiling Consumers Who Are Ready to Adopt Insects as a Meat Substitute in a Western Society. Food Qual. Prefer..

[28] Wassmann B., Siegrist M., Hartmann C. (2021). Correlates of the Willingness to Consume Insects: A Meta-Analysis. J. Insects Food Feed.

[29] De Ramírez-Rivera E.J., Cabal-Prieto A., Gómez-Romero E., Oney-Montalvo J.E., Can-Herrera L.A., Hernández-Salinas G., Sánchez-Orea J.M., Valdivia-Sánchez J., Martínez A.L.P., de Reséndiz J.J.G. (2025). Challenges to Insect-Based Food Acceptance: An Analysis of Neophobia Exploring Cognitive Aspects of the Mexican Consumers. J. Food Sci..

[30] Glanz K., Basil M., Maibach E., Goldberg J., Snyder D. (1998). Why Americans Eat What They Do. J. Am. Diet. Assoc..

[31] Cornell C.E., Rodin J., Weingarten H. (1989). Stimulus-Induced Eating When Satiated. Physiol. Behav..

[32] Marcelino A.S., Adam A.S., Couronne T., Köster E.P., Sieffermann J.M. (2001). Internal and External Determinants of Eating Initiation in Humans. Appetite.

[33] Beaver J.D., Lawrence A.D., van Ditzhuijzen J., Davis M.H., Woods A., Calder A.J. (2006). Individual Differences in Reward Drive Predict Neural Responses to Images of Food. J. Neurosci..

[34] Stoeckel L.E., Cox J.E., Cook E.W., Weller R.E. (2007). Motivational State Modulates the Hedonic Value of Food Images Differently in Men and Women. Appetite.

[35] Hurling R., Shepherd R. (2003). Eating with Your Eyes: Effect of Appearance on Expectations of Liking. Appetite.

[36] Jansen E., Mulkens S., Jansen A. (2010). How to Promote Fruit Consumption in Children. Visual Appeal versus Restriction. Appetite.

[37] Zellner D.A., Siemers E., Teran V., Conroy R., Lankford M., Agrafiotis A., Ambrose L., Locher P. (2011). Neatness Counts. How Plating Affects Liking for the Taste of Food. Appetite.

[38] Piqueras-Fiszman B., Alcaide J., Roura E., Spence C. (2012). Is It the Plate or Is It the Food? Assessing the Influence of the Color (Black or White) and Shape of the Plate on the Perception of the Food Placed on It. Food Qual. Prefer..

[39] Nikravech M., Cubero Dudinskaya E., Rumpold B.A., de Almeida Costa A.I., Zanoli R., Langen N. (2025). The Role of Prior Information on Consumer Acceptance of Insect-Based Food and Feed in Europe: Evidence from a Discrete Choice Experiment. Future Foods.

[40] Moruzzo R., Riccioli F., Espinosa Diaz S., Secci C., Poli G., Mancini S. (2021). Mealworm (*Tenebrio molitor*): Potential and Challenges to Promote Circular Economy. Animals.

[41] Grispoldi L., Zampogni L., Costanzi E., Karama M., El-Ashram S., Al-Olayan E., Saraiva C., Garcia-Diez J., Iulietto M.F., Cenci-Goga B. (2023). Exploring Consumer Perception of Entomophagy by Applying the Rasch Model: Data from an Online Survey. J. Insects Food Feed.

[42] Buongiovanni R., Pisano M.T., Spagnolo F.M.A., Locorriere D., Lorini C., Del Riccio M., Bonaccorsi G. (2025). Insect-Based Novel Food: Is Italy Ready for the Food of the Future? A Survey on Entomophagy Among Italian People. Ann. Ig..

[43] Ting H., Memon M.A., Thurasamy R., Cheah J.-H. (2025). Snowball Sampling: A Review and Guidelines for Survey Research. Asian J. Bus. Res..

[44] Tuccillo F., Marino M.G., Torri L. (2020). Italian Consumers’ Attitudes towards Entomophagy: Influence of Human Factors and Properties of Insects and Insect-Based Food. Food Res. Int..

[45] Serpico M., Rovai D., Wilke K., Lesniauskas R., Garza J., Lammert A. (2021). Studying the Emotional Response to Insects Food Products. Foods.

[46] Turck D., Bohn T., Castenmiller J., De Henauw S., Hirsch-Ernst K.I., Maciuk A., Mangelsdorf I., McArdle H.J., Naska A., Pelaez C. (2021). Safety of Frozen and Dried Formulations from Whole House Crickets (*Acheta domesticus*) as a Novel Food Pursuant to Regulation (EU) 2015/2283. EFSA J..

[47] Sanz T., Salvador A., Fiszman S.M. (2008). Evaluation of Four Types of Resistant Starch in Muffin Baking Performance and Relationship with Batter Rheology. Eur. Food Res. Technol..

[48] Baldino N., Paleologo M.F.O., Chiodo M., Mileti O., Lupi F.R., Gabriele D. (2025). Assisted Extraction of Hemp Oil and Its Application to Design Functional Gluten-Free Bakery Foods. Molecules.

[49] ISTAT Istituto Nazionale di Statistica Censimento e Dinamica Della Popolazione—Anno 2024. https://www.istat.it/.

[50] Tavakol M., Dennick R. (2011). Making Sense of Cronbach’s Alpha. Int. J. Med. Educ..

[51] Norman G. (2010). Likert Scales, Levels of Measurement and the “Laws” of Statistics. Adv. Health Sci. Educ..

[52] Carifio J., Perla R. (2008). Resolving the 50-year Debate around Using and Misusing Likert Scales. Med. Educ..

[53] Taber K.S. (2018). The Use of Cronbach’s Alpha When Developing and Reporting Research Instruments in Science Education. Res. Sci. Educ..

[54] Errico S., Mastrobuono V., Pagliarello R., Bennici E., Tavazza R., Verardi A., Presenti O., Panozzo M., Sangiorgio P., Massa S. (2025). Consumer Acceptance of Edible Hydrogels Obtained by Plant Cell Culture Technology and By-Products Valorization: An Italian Case Study for Future Innovation of the Plate. Innov. Food Sci. Emerg. Technol..

[55] Sangiorgio P., Errico S., Verardi A., Massa S., Pagliarello R., Marusic C., Lico C., Presenti O., Donini M., Baschieri S. (2023). Consumer Awareness and Acceptance of Biotechnological Solutions for Gluten-Free Products. Foods.

[56] Smith W.G. (2008). Does Gender Influence Online Survey Participation? A Record-Linkage Analysis of University Faculty Online Survey Response Behavior.

[57] Panchendrarajan R., Saxena H., Saxena A. (2025). Social Media and Academia: How Gender Influences Online Scholarly Discourse. arXiv.

[58] Tolve R., Zanoni M., Sportiello L., Musollini S., Tchuenbou-Magaia F.L., Favati F. (2025). From Fear to Fork—Exploring Food Neophobia and the Inclination towards Entomophagy in Italy. Int. J. Food Sci. Technol..

[59] Hartmann C., Siegrist M. (2017). Consumer Perception and Behaviour Regarding Sustainable Protein Consumption: A Systematic Review. Trends Food Sci. Technol..

[60] Gmuer A., Nuessli Guth J., Hartmann C., Siegrist M. (2016). Effects of the Degree of Processing of Insect Ingredients in Snacks on Expected Emotional Experiences and Willingness to Eat. Food Qual. Prefer..

[61] Rehman N., Ogrinc N. (2024). Consumer Perceptions and Acceptance of Edible Insects in Slovenia. Foods.

[62] Cicatiello C., Vitali A., Lacetera N. (2020). How Does It Taste? Appreciation of Insect-Based Snacks and Its Determinants. Int. J. Gastron. Food Sci..

[63] Arena E., Mazzaglia A., Selvaggi R., Pecorino B., Fallico B., Serranò M., Pappalardo G. (2020). Exploring Consumer’s Propensity to Consume Insect-Based Foods. Empirical Evidence from a Study in Southern Italy. Appl. Syst. Innov..

[64] Lammers P., Ullmann L.M., Fiebelkorn F. (2019). Acceptance of Insects as Food in Germany: Is It about Sensation Seeking, Sustainability Consciousness, or Food Disgust?. Food Qual. Prefer..

[65] Hartmann C., Siegrist M. (2017). Insects as Food: Perception and Acceptance. Ernaehrungs Umsch. Int..

[66] Alhujaili A., Nocella G., Macready A. (2023). Insects as Food: Consumers’ Acceptance and Marketing. Foods.

[67] Abro Z., Sibhatu K.T., Fetene G.M., Alemu M.H., Tanga C.M., Sevgan S., Kassie M. (2025). Global Review of Consumer Preferences and Willingness to Pay for Edible Insects and Derived Products. Glob. Food Sec..

[68] Syartiwidya, Iwansyah A.C., Fikri A.M., Harianti R., Yunita R., Saghita E.P. (2025). A Global Perspective and Acceptability of Edible Insects as Novel Food in Western and Asian Countries: A Systematic Literature Review. Food Prod. Process. Nutr..

[69] Ros-Baró M., Sánchez-Socarrás V., Santos-Pagès M., Bach-Faig A., Aguilar-Martínez A. (2022). Consumers’ Acceptability and Perception of Edible Insects as an Emerging Protein Source. Int. J. Environ. Res. Public Health.

