# Supplementary material for: Visual Assessment and Information Effects on Consumer Acceptance of Insect-Based Foods: The Role of Attitudes, Knowledge, and Sociodemographics
Source: Foods. 2026 May 12;15(10):1703. doi: 10.3390/foods15101703 (PMC13205508; doi:10.3390/foods15101703)
Supplement: Supplementary file 1 [file foods-15-01703-s001.zip › foods-4268778-supplementary.pdf]

SUPPLEMENTARY MATERIALS

Visual Assessment and Information Effects on Consumer Acceptance of Insect-Based Foods: The Role of Attitudes, Knowledge, and Sociodemographics

Alessandra Verardi[1,†,*], Paola Sangiorgio[1,†], Olga Mileti[2], Mariateresa Chiodo[2], Noemi Baldino[2], and Simona Errico[1]

SECTION S1
QUESTIONNAIRE

Dear Consumer,
This questionnaire is part of exploratory activities conducted within the collaboration between ENEA and the University of Calabria (UNICAL).
The questionnaire consists of 10 questions and will take approximately 5 minutes to complete. It is strictly anonymous and aims solely to collect insights into consumer perceptions from a statistically relevant sample. Therefore, it will not be used for any other purpose, and there are no right or wrong answers; only your personal opinion is required.

Processing of Responses
All responses will remain anonymous and confidential. Results will be reported in aggregated form, ensuring that no individual participant can be identified. Raw data will be securely stored, and access to such data, including any potentially identifiable information, will be restricted to a single authorized and identified ENEA researcher.

Participation in the Survey
Participation in this survey is voluntary, involves no personal obligations, and participants may withdraw at any time by closing the browser and not submitting the questionnaire.

Electronic Informed Consent
By clicking the "Accept" button, you confirm that:
- you have read and understood the information provided above
- you voluntarily agree to participate in this survey

If you agree to participate, please click "Accept" to proceed.
If you do not wish to participate, please click "Do not accept".

The ENEA and UNICAL research team sincerely thank you for your cooperation, availability, and the time you are willing to dedicate.

(*) Mandatory

Consent to participate (*)
- Accept
- Decline

1- **Region of residence (*)**
- ❑ Abruzzo
- ❑ Basilicata
- ❑ Calabria
- ❑ Campania
- ❑ Emilia-Romagna
- ❑ Friuli-Venezia Giulia
- ❑ Lazio
- ❑ Liguria
- ❑ Lombardy
- ❑ Marche
- ❑ Molise
- ❑ Piedmont
- ❑ Apulia
- ❑ Sardinia
- ❑ Sicily
- ❑ Tuscany
- ❑ Trentino-Alto Adige
- ❑ Umbria
- ❑ Aosta Valley
- ❑ Veneto

2- **Gender (*)**
- ❑ Male
- ❑ Female
- ❑ I prefer not to specify.

3- **Age (*)**
- ❑ Under 18
- ❑ 19–30
- ❑ 31–50
- ❑ 51–64
- ❑ 65 or older

4- **Education level (*)**
- ❑ None
- ❑ Primary school
- ❑ Lower secondary school
- ❑ High school diploma
- ❑ Bachelor's degree
- ❑ Master's degree (5 years or more)
- ❑ PhD / Specialization / Postgraduate Master

5- **Background (*)**
- ❑ Humanities
- ❑ Scientific
- ❑ Not sure

6- **Do you usually consume soft baked sweet products (e.g., muffins, cakes, brioche, cupcakes)? (*)**
(1 = never; 2 = rarely; 3 = sometimes; 4 = often; 5 = daily)

1	2	3	4	5

7- Look at the picture of this food: how appealing do you find it? (*)

(1 = not at all; 2 = slightly; 3 = neutral; 4 = fairly; 5 = very)

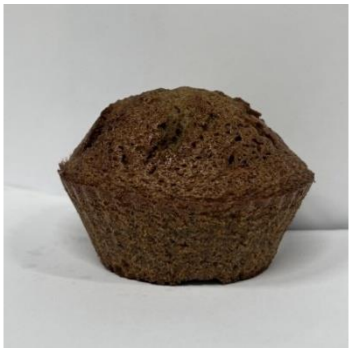
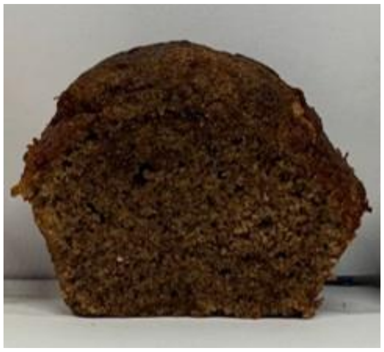

1	2	3	4	5

8- The product shown in the image was made using 10% flour derived from larvae of *Tenebrio molitor*, an insect approved by the European Commission for human consumption. Now that you are aware of this, please reassess its appeal. (*)

(1 = not at all; 2 = slightly; 3 = neutral; 4 = fairly; 5 = very)

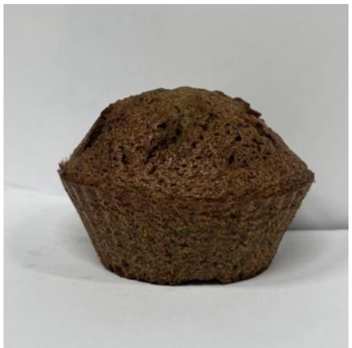
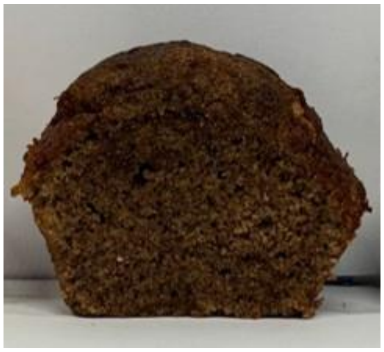

1	2	3	4	5

9- Please indicate your level of agreement: (*)

(1 = not at all; 2 = slightly; 3 = neutral; 4 = moderately; 5 = very)

		1	2	3	4	5
1	It is unlikely that insect-based products will be successful in Europe					
2	I would feel uncomfortable consuming food containing insect flour					
3	I believe that insect-based foods may become common in the future					
4	I am concerned that consuming insect-based foods may be harmful to health					

5	I believe that insect-based foods can be safe if produced under controlled conditions					
6	I would taste this product out of curiosity					
7	My curiosity to try a product strongly depends on its appearance					
8	Even if the appearance is appealing, the idea that it contains insect flour would prevent me from tasting it					
9	Even if the appearance is appealing, I am concerned that I might not like its taste					
10	I would consume it to diversify my protein sources					
11	I would try it if the price were lower					

10- Please indicate how familiar you are with the following items: (*)

(1 = not at all; 2 = slightly; 3 = neutral; 4 = moderately; 5 = very)

		1	2	3	4	5
1	Insect flour can be used to produce a variety of food products (e.g., pasta, crackers, sweet and savory snacks)					
2	The European Commission has authorized the use of four insect species in powdered form					
3	The European Commission allows the consumption of whole insects (if dried or frozen) belonging to authorized species					
4	National Regulation states the insect flour must not exceed 10% of the total ingredients					
5	Global protein demand will increase in the coming years due to population growth					
6	Insects provide an alternative protein source to conventional livestock (e.g., cattle, sheep, poultry, fish)					
7	Insects contain high-quality proteins, essential amino acids, and important nutrients such as vitamins and minerals					
8	Insect farming results in lower greenhouse gas emissions, lower water and land use compared to conventional livestock farming					

Thank you for your cooperation

SECTION S2

Table S1. Component loadings for the 19 attitudinal (ATT) and knowledge-related (KNO) items included in the Principal Component Analysis (PCA), as described in Section 3.6. Loadings indicate the strength and direction of the association between each item and the two extracted components (PC1 and PC2). Higher absolute values represent stronger contributions to the underlying latent dimensions.

**Item**	**PC1**	**PC2**
It is unlikely that insect-based products will be successful in Europe	-0.02917	0.207531
I would feel uncomfortable consuming food containing insect flour	-0.15779	0.380955
I believe that insect-based foods may become common in the future	0.22381	-0.05434
I am concerned that consuming insect-based foods may be harmful to health	-0.16777	0.234549
I believe that insect-based foods can be safe if produced under controlled conditions	0.248556	-0.11239
I would taste this product out of curiosity	0.231901	-0.24878
My curiosity to try a product strongly depends on its appearance	0.033139	-0.01302
Even if the appearance is appealing, the idea that it contains insect flour would prevent me from tasting it	-0.12772	0.374765
Even if the appearance is appealing, I am concerned that I might not like its taste	-0.07372	0.334052
I would consume it to diversify my protein sources	0.235819	-0.2805
I would try it if the price were lower	0.224594	-0.25518
